# Clinical characteristics and outcomes of discharged COVID-19 patients with reoccurrence of SARS-CoV-2 RNA

**DOI:** 10.2217/fvl-2020-0142

**Published:** 2020-10-22

**Authors:** Jing Wu, Xiao-ying Xia, He-lei Liu, Hong Xia, Wen-xiang Huang, Bei Jia, Feng-ying Peng

**Affiliations:** 1 Department of Infectious Disease, The People’s Hospital of Dazu District, Chongqing 402360, China; 2 Key Laboratory of Infectious & Parasitic Diseases in Chongqing, Department of Infectious Disease, The First Affiliated Hospital of Chongqing Medical University, Chongqing 400016, China

**Keywords:** clinical characteristics, COVID-19, discharge, outcomes, reoccurrence, SARS-CoV-2 RNA

## Abstract

**Aim:**

Data are limited on clinical characteristics and outcomes of recovered the 2019 coronavirus disease (COVID-19) patients with the reoccurrence of SARS-CoV-2 RNA.

**Patients & methods:**

Discharged patients in our hospital were included, who had recovered from COVID-19 with the reoccurrence of SARS-CoV-2 RNA.

**Results:**

Six patients were redetectable and positive for SARS-CoV-2 RNA after discharge from 3 to 15 days. The main symptoms, although no fever, included fatigue, dry cough and pharyngeal or chest discomfort, which were generally milder in the repositive period compared with the period of initial infection. Their laboratory indexes were significantly improved compared with the initial infection, and the pulmonary lesions were continuously improving. All close contacts were SARS-CoV-2 RNA-negative.

**Conclusion:**

No worsening outcomes or active transmission to close contacts were found for the repositive COVID-19 patients.

With the gradual control of the outbreak of the 2019 coronavirus disease (COVID-19) in China, nearly 92% (75,949/82,505) [1] of patients have clinically recovered and been discharged from hospital. Attention should be focused on this group of people regarding the possibility of reemerging clinical manifestations and infectivity. One published investigation [2] found that SARS-CoV-2 RNA was detected again around 1 week after patients were discharged with twice-negative reverse transcriptase-polymerase chain reaction (RT-PCR) test results; and so did the follow-up of 262 discharged patients in Guangdong Province, China, found that approximately 14.5% were positive for SARS-CoV-2 RNA. However, few studies have reported the detailed progression of these patients in terms of their clinical characteristics, laboratory parameters, imaging findings and transmission possibility. In this study, we analyzed COVID-19 patients who underwent treatment and had recovered and were positive again by RT-PCR for SARS-CoV-2 RNA after discharge in a county hospital of western Chongqing, China. Here, we described the clinical characteristics and outcomes of these patients.

## Patients & methods

### Patients

Discharged patients who had recovered from COVID-19 with the reoccurrence of SARS-CoV-2 RNA from 23 January 2020 to 30 March 2020 were included.

## Methods

### Data collection

The clinical data of patients with the reoccurrence of SARS-CoV-2 RNA were collected in the People’s Hospital of Dazu District, western Chongqing, China. Their demographic data, epidemiological characteristics, clinical manifestations, laboratory results, chest computed tomography (CT) imaging findings and therapeutic drug information were collected.

### Detection reagents

Novel Coronavirus (2019-nCoV) Nucleic Acid Diagnostic Kit (PCR-Fluorescence probing, Sansure Biotech Limited Corporation, Changsha City, China) was used to detect viral nucleic acid in samples from the nose, throat and anus of the included patients. A SARS-CoV-2 IgM and IgG antibody quantitative detection kit (chemiluminescence, YHLO Biotech Limited Corporation, Shenzhen City, China) was used to detect serum viral antibodies.

### Criteria for test results

For viral nucleic acid detection, oropharyngeal, nasopharyngeal and anal swabs of patients were collected by trained qualified nurses, and the open-reading frame 1ab (*ORF1ab*) and nucleocapsid protein (*N*) genes were detected using real-time fluorescence RT-PCR. The result was positive under the following conditions: the typical S-type amplification curve of the *ORF1ab* and *N* genes in the same sample was detected simultaneously, and the amplification cycle threshold (Ct) was less than 40. For the detection of virus IgM and IgG antibodies, the serum samples of all patients were detected by SARS-CoV-2 IgM and IgG antibody detection kits. When the concentration of IgM and IgG antibodies was less than 10·0 AU/ml, the result was considered nonreactive (negative); when the concentration of IgM and IgG antibodies was ≥10·0 AU/ml, the result was considered reactive (positive).

### Criteria for diagnosis & discharge

Referring to the Novel Coronavirus Infected Pneumonia Treatment Program, Trial version 6, China [3], the following discharge criteria were applied: body temperature returned to normal for more than 3 days; respiratory symptoms significantly improved; pulmonary CT imaging showed that acute exudative lesions were significantly improved; and a nucleic acid test of respiratory tract samples was negative twice in succession (sampling interval time was at least 1 day).

### Statistical methods

SPSS version 20.0 software (IBM Corp, NY, USA) was used for the analyses. Normally distributed measurement data were described as mean ± standard deviation, and non-normally distributed measurement data were expressed as median (range). Count data were expressed as frequency (percentage).

## Results

### General information

A total of six patients were included, five men (83·3%) and one woman. The average age was 50.83 years (±23.83 years), and the median age was 46 years (range: 32–71). The patients were farmers or workers. Patient 5 had a history of living in Wuhan, and the other five patients were infected by close contact with confirmed patients. The average duration of disease was 4.83 days (±2.86 days), and the median disease duration was 4 days at the first admission. Each two of these six patients at first admission was classified as mild, and moderate and severe type. The longest incubation period was 13 days, and the average incubation period was 6.7 days (±4.1 days). Two patients (33·33%) had co-morbidities; the remainder had been in good health previously (Table 1).

**Table 1.** Basic information of patients and co-morbidities.

Case	Occupation	Age (y)/sex	Epidemiological history	Clinical classification	Co-morbidities	Incubation period (days)	Disease course at first admission (days)
1	Farmer	71/F	Contact with confirmed patients	Severe	No	4	2
2	Farmer	39/M	Contact with confirmed patients	Mild	No	10	4
3	Farmer	44/M	Contact with confirmed patients	Moderate	Hypertension	13	3
4	Worker	71/M	Contact with confirmed patients	Mild	Hypertension, liver cirrhosis	6	6
5	Farmer	32/M	History of living in Wuhan	Moderate	No	5	4
6	Worker	48/M	Contact with confirmed patients	Severe	No	2	10

### Clinical manifestations & treatment

At the first admission, the main symptom was dry cough (83.33%, 5/6) followed by fatigue (50%, 3/6), dry pharynx (1/6), pharyngeal itching (1/6) and chest tightness (1/6) with different severity. Patient 4 was asymptomatic. The average length of hospital stay was 14.8 days (median: 12 days [range: 7–25]).

During hospitalization after the reoccurrence of SARS-CoV-2 RNA, the following symptoms were noted: dry cough (33.33%, 2/6), diarrhea (1/6) and runny nose (1/6). All the symptoms were mild, and the average length of hospital stay was 11.7 days (median: 11.5 days [range: 7–20]).

The main drugs used during the first hospitalization period were atomized IFN-α-1b 60 μg b.i.d.; oral administration of Lopinavir/rotanavir (LPV + RTV) 500 mg b.i.d.; thymopentin (TP5) 10 mg im qod; and traditional Chinese medicine. The drugs used after the reoccurrence of SARS-CoV-2 RNA were atomized IFN-α-1b 60 μg b.i.d.; traditional Chinese medicine; hydroxychloroquine (HCQ) 600 mg on the first day, 400 mg after 6 h and 400 mg q.d. on the second day for 10 days; and TP5 10 mg im. q.o.d. Notably, patient 4 had anxiety, and the Sleep Psychosomatic Center was consulted for psychological counseling (Table 2).

**Table 2.** Clinical condition at first admission and after reoccurrence of SARS-CoV-2 RNA.

Case	Symptoms at first admission	Symptoms at RSR	Drugs used at first admission	Drugs used after RSR	LOS of first admission (days)	LOS after RSR (days)
1	Dry cough	None	*IFN-α-1b, LPV + RTV*	IFN-α-1b	8	7
2	Fatigue, chest tightness, dry cough	Diarrhea	LPV + RTV, IFN-α-1b	Traditional Chinese medicine	9	7
3	Dry cough	Dry cough and runny rose	IFN-α-1b, LPV + RTV	Traditional Chinese medicine, HCQ, IFN-α-1b	7	20
4	None	Dry cough, anxiety	LPV + RTV, IFN-α-1b	IFN-α-1b, Traditional Chinese medicine, TP5	25	7
5	Dry cough, fatigue, dry pharynx, pharyngeal itching	None	IFN-α-1b, traditional Chinese medicine, LPV + RTV, TP5	TP5, HCQ, Traditional Chinese medicine	25	16
6	Dry cough, fatigue	None	IFN-α-1b, LPV + RTV	HCQ, IFN-α-1b, TP5, Traditional Chinese medicine	15	16

HCQ: Hydroxychloroquine; IFN: Interferon; LOS: Length of hospital stay; LPV + RTV: Lopinavir + ritonavir; RSR: Reoccurrence of SARS-CoV-2 RNA; TP5: Thymopentin.

### Comparison of laboratory examination indexes before & after the reoccurrence of SARS-CoV-2 RNA

At the first admission, the mean values of the lymphocyte percentage and absolute value of lymphocytes decreased, the average values of C-reactive protein and the erythrocyte sedimentation rate increased, the mean values of procalcitonin were normal, the CD4^+^ and CD8^+^ lymphocyte counts decreased in varying degrees, and the average values of aspartate aminotransferase and creatine kinase isoenzyme increased slightly; however, the average values of these indexes were all in the normal range after the reoccurrence of SARS-CoV-2 RNA. Respiratory tract virus antigens were negative before and after the reoccurrence of SARS-CoV-2 RNA (Table 3).

**Table 3.** Changes in laboratory indexes before and after the reoccurrence of SARS-CoV-2 RNA.

Indexes	Initial hospitalization	After RSR
WBC (3.5–9.5 × 10^9^/l)	6.17 ± 2.08	4.94 ± 1.17
Lymph% (20–50)	17.67 ± 10.56	26.02 ± 5.88
Lymph^#^ (1.1–3.2 × 10^9^/l)	1.04 ± 0.38	1.33 ± 0.52
PLT (100–400 × 10^9^/l)	159.33 ± 66.90	188.50 ± 93.21
ALT (9–50 U/l)	39.03 ± 17.57	47.03 ± 16.20
AST (15–40 U/l)	41.15 ± 22.38	24.53 ± 9.60
ALB (40–55 g/l)	43.83 ± 4.80	42.95 ± 3.30
CD8^+^ (144–699 cells/μl)	87–517	53–595
CD4^+^ (355–1213 cells/μl)	61–652	233–785
PO_2_ (80–100 mmHg)	104.5 ± 23.32	104.75 ± 34.02
LAC (mmol/l)	2.00 ± 0.99	2.19 ± 0.57
CRP (0–3.3 mg/l)	18.32 ± 22.49	0.6 ± 0.42
ESR (0–15 mm/h)	23.33 ± 16.33	17.83 ± 12.42
PCT (0–0.5 ng/ml)	0.21 ± 0.14	0.17 ± 0.06
Cr (62–115 μmol/l)	72.6 ± 17.04	81.64 ± 12.63
Urea (3.1–8 mmol/l)	4.96 ± 1.05	5.28 ± 1.29
CK (50–310 U/l)	123.92 ± 84.38	58.97 ± 13.18
CK-MB (0–24 U/l)	20.03 ± 13.48	15.70 ± 11.96
PT (9–14 s)	10.37 ± 1.19	10.33 ± 1.09
Respiratory tract virus antigens	Negative	Negative
Influenza A/B virus antigen	Negative	Negative

ALB: Albumin; ALT: Alanine aminotransferase; AST: Aspartate aminotransferase; CK: Creatine kinase; CK-MB: Creatine kinase isoenzyme; Cr: Creatinine; CRP: C-reactive protein; ESR: Erythrocyte sedimentation rate; LAC: Lactic acid; Lymph^#^: Absolute value of lymphocytes; Lymph%: Lymphocyte percentage; PCT: Procalcitonin; PLT: Platelets; PO_2_: Partial pressure of oxygen; PT: Prothrombin time; RSR: Reoccurrence of SARS-CoV-2 RNA; Urea: Urea nitrogen; WBC: White blood cell.

### Isolation measures & SARS-CoV-2 RNA detection after the first discharge

All six patients were isolated at home after clinical recovery and discharge; however, they were required to return to hospital every 3 days for SARS-CoV-2 RNA detection tests. Once one of the six patients had a positive result, all the six discharged patients were immediately centrally isolated; the shortest time to the reoccurrence of SARS-CoV-2 RNA was 3 days, and the longest was 15 days (average 6.5 days). For all patients with the reoccurrence of SARS-CoV-2 RNA, oropharyngeal, nasopharyngeal and anal swabs were simultaneously obtained at intervals of more than 24 h; the lowest number of samples taken was four-times, and the highest was 13 (median seven-times). The RT-PCR Ct values were all less than 40 but approximated 40. The patients could not be discharged until the virus nucleic acid test was negative two or more times consecutively. The shortest time to SARS-CoV-2 RNA-negative conversion after readmission was 1 day, and the longest was 16 days (median: 7 days; average 7.5 days). All the patients were tested for viral IgG and IgM antibodies at readmission and before redischarge, and the results were all positive for IgG antibodies and negative for IgM antibodies (Table 4).

**Table 4.** Quarantine measures and viral nucleic acid detection after the reoccurrence of SARS-CoV-2 RNA.

Case no.				1	2	3	4	5	6
Quarantine measures				Home	Home	Home	Centralized	Home and centralized	Home and centralized
Time of RSR, days				6	3	3	6	15	6
Viral nucleic acid detection after RSR	First	PCR, Ct^†^	NPS	37.51, 37.41	38.93, 39.86	Negative	37.52, 37.67	Negative	Negative
			OPS	Negative	38.31, 37.32	Negative	38.36, 38.52	38.05, 39.49	Negative
			Anal	Negative	Negative	37.69, 38.40	Negative	Negative	36.47, 37.91
		IgG (AU/ml)		156.9	26.65	72.12	159.02	180.82	118.79
		IgM (AU/ml)		7.83	0.58	1.46	5.11	2.32	7.09
	Second	PCR, Ct	NPS	Negative	Negative	Negative	Negative	Negative	Negative
			OPS	Negative	Negative	39.90, 38.02	Negative	Negative	Negative
			Anal	Negative	Negative	37.56, 38.24	Negative	Negative	38.89, 39.01
	Third	PCR, Ct	NPS	Negative	Negative	Negative	Negative	Negative	Negative
			OPS	Negative	Negative	Negative	Negative	Negative	Negative
			Anal	Negative	Negative	39.62, 38.18	Negative	Negative	38.32, 39.56
	Fourth	PCR, Ct	NPS	Negative	Negative	37.29, 38.16	Negative	38.87, 39.24	Negative
			OPS	Negative	Negative	Negative	Negative	Negative	Negative
			Anal	Negative	Negative	38.71, 38.52	Negative	Negative	Negative
		IgG (AU/ml)		124.5	52.3	–	181.62	–	-
		IgM (AU/ml)		2.63	0.55	–	4.34	–	-
	Fifth	PCR, Ct	NPS	–	–	Negative	–	Negative	Negative
			OPS	–	–	Negative	–	38.26, 37.36	Negative
			Anal	–	–	Negative	–	Negative	Negative
	Sixth	PCR, Ct	NPS	–	–	36.34, 37.37	–	Negative	38.91, 39.97
			OPS	–	–	Negative	–	Negative	Negative
			Anal	–	–	38.21, 37.97	–	Negative	Negative
	Seventh	PCR, Ct	NPS	–	–	Negative	–	Negative	Negative
			OPS	–	–	37.70, 39.28	–	38.76, 39.13	Negative
			Anal	–	–	39.22, 39.36	–	Negative	Negative
	Eighth	PCR, Ct	NPS	–	–	Negative	–	Negative	Negative
			OPS	–	–	38.94, 37.86	–	Negative	Negative
			Anal	–	–	Negative	–	Negative	Negative
	Ninth	PCR, Ct	NPS	–	–	Negative	–	Negative	36.77 37.01
			OPS	–	–	36.67 35.43	–	Negative	Negative
			Anal	–	–	Negative	–	Negative	Negative
	Tenth	PCR, Ct	NPS	–	–	Negative	–	Negative	Negative
			OPS	–	–	39.16 39.65	–	Negative	Negative
			Anal	–	–	Negative	–	Negative	Negative
		IgG (AU/ml)		–	–	–	–	145.09	-
		IgM (AU/ml)		–	–	–	–	1.46	-
	Eleventh	PCR, Ct	NPS	–	–	Negative	–	–	Negative
			OPS	–	–	Negative	–	–	Negative
			Anal	–	–	Negative	–	–	Negative
	Twelfth	PCR, Ct	NPS	–	–	Negative	–	–	Negative
			OPS	–	–	Negative	–	–	Negative
			Anal	–	–	Negative	–	–	Negative
		IgG (AU/ml)		–	–	–	–	–	194.26
		IgM (AU/ml)		–	–	–	–	–	4.85
			IgM (AU/ml)	–	–	–	–	–	4.85
	Thirteenth	PCR, Ct	NPS	–	–	Negative	–	–	-
			OPS	–	–	Negative	–	–	-
			Anal	–	–	Negative	–	–	-
		IgG (AU/ml)		–	–	105.23	–	–	-
		IgM (AU/ml)		–	–	1.13	–	–	-

† PCR, Ct: The first number in the cell denotes the value of the *ORF1ab* gene cycle threshold; the second number denotes the values of the *N* gene cycle threshold.

Ct: Cycle threshold; Ig: Immunoglobulin; NPS: Nasopharyngeal swab; OPS: Oropharyngeal swab; PCR: Polymerase chain reaction; RSR: Reoccurrence of SARS-CoV-2 RNA.

### Imaging findings

**Figure 1.**
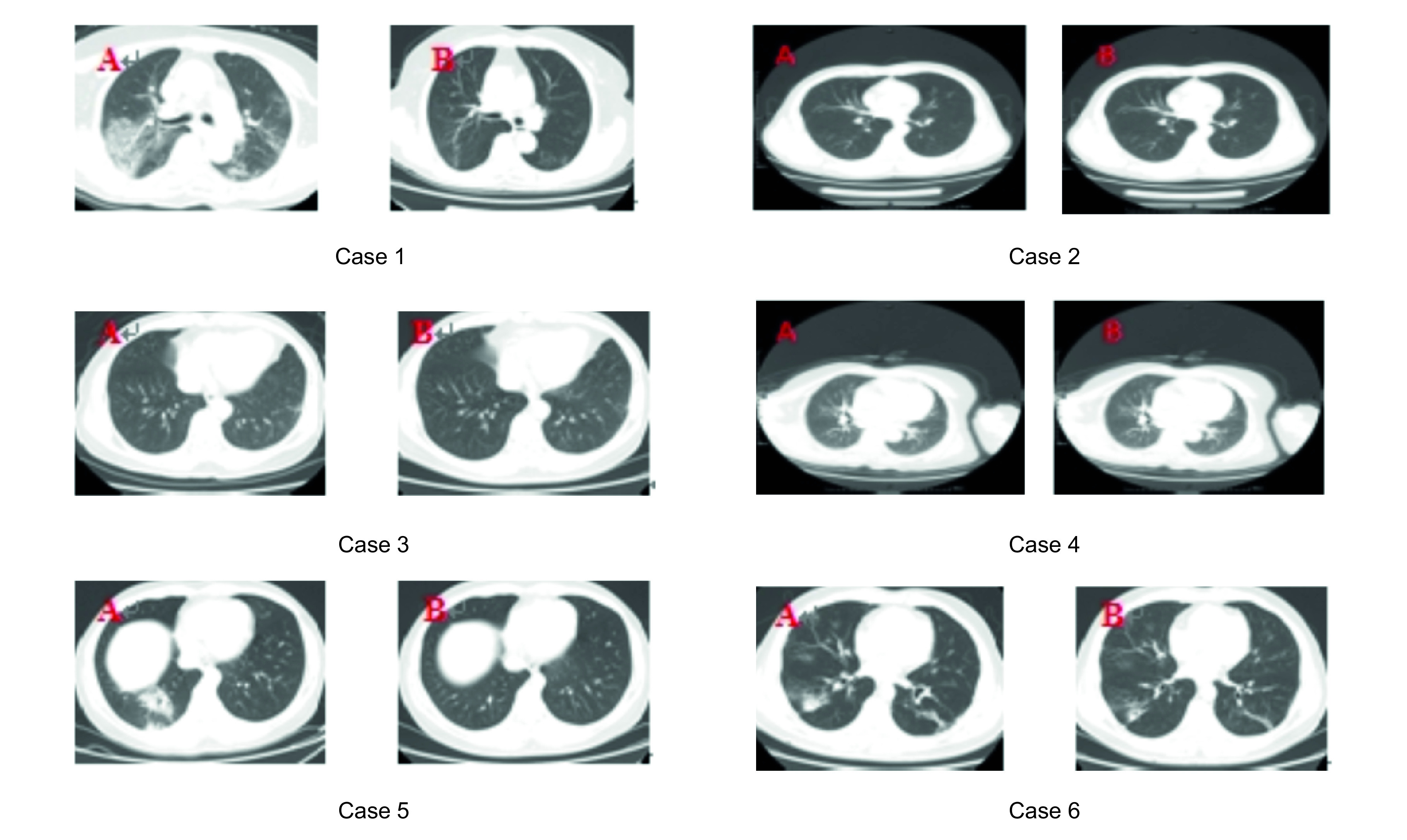
Chest computed tomography findings of all six cases in this study. **(A)** Chest CT image at first admission. **(B)** Chest CT image after the reoccurrence of SARS-CoV-2 RNA. CT: Computed tomography.

Six patients underwent chest CT examination after the first admission and reoccurrence of SARS-CoV-2 RNA. Patient 1 had multiple patchy shadows in both lungs involving the stroma, and patient 2 showed no abnormality on chest CT. Patient 3 showed a small flocculent-blurred shadow in the outer basal segment of the left lower lobe near the subpleural space, and patient 4 showed no abnormality on chest CT. Patient 5 showed nodular and patchy ground-glass density in the basal segment of the lower lobe of the right lung and the sublingual segment of the upper lobe of the left lung, and patient 6 showed multiple patchy light shadows and fibrous strips in both lungs (Figure 1). Generally, no progression of the lesions was found for all these patients comparing to those prior imaging.

## Discussion

A previous study [2] reported that patients under the age of 14 years with the reoccurrence of SARS-CoV-2 RNA account for 35% of their age group after discharge, suggesting that young people are prone to experience the reoccurrence of SARS-CoV-2 RNA. In our cases, the average age of the six patients in our study was 50.83 years old suggesting that follow-up management should be implemented for patients of all ages. Among the six patients, only one is female, but it is not universal whether males with COVID-19 infection are more likely to experience the reoccurrence of SARS-CoV-2 RNA, which needs to be confirmed by a large sample size study. Among the six patients, only one had a history of living in Wuhan, and the other five were infected by contact with confirmed cases in accordance with the early epidemic characteristics of the disease. It has been reported [2,4] that patients with redetectable RNA experience the mild–moderate clinical type with mild symptoms; however, in our study, two cases were mild type, two were common type and two were severe type suggesting that patients should be followed up after discharge regardless of their clinical type. The explanation for the reoccurrence of SARS-CoV-2 RNA in these patients might be the potential long-term effect of drugs or diseases that hamper the immune response [5].

In the early stage of the disease, the main clinical manifestations were cough and fatigue in all six patients that were consistent with a study including 138 patients diagnosed with COVID-19 [6]; interestingly, none of the patients in our study had a fever whether they were in hospital or at home, suggesting the importance of wearing masks and social distancing in infection control. After the redetection of SARS-CoV-2 RNA, only two patients had a cough, one had diarrhea and one had a runny nose; three cases were asymptomatic; therefore, the symptoms were mild. One of the patients may have had a cough and runny nose after catching a cold. These findings indicate that the conditions of most patients were continuously improving even though they retested positive for SARS-CoV-2 RNA.

There was no difference in the hospitalization time before and after the reoccurrence of SARS-CoV-2 RNA; the median hospitalization time was approximately 12 days. Currently, the medication we were using was based on the experiences of treating SARS and some findings of *in vitro* experiments. HCQ was used for three patients with the reoccurrence of SARS-CoV-2 RNA. HCQ exerts an antiviral effect by changing the pH of the endosome, inhibiting viral gene expression and acting as an autophagy inhibitor [7]. But we did not see any significant improvement in these three cases. Although no adverse reactions were observed after the use of the aforementioned drugs, caution must be taken because multiple drugs were used and drug–drug interaction should be looked out for. The combined average hospitalization time of the two admissions was 27 days, suggesting that the persistence possibility of virus in the body is likely to be within 1 month. As a result of the long isolation treatment time in hospital, one patient had symptoms of psychological anxiety. One study has shown that patients with COVID-19 have obvious depression and anxiety as well as various somatic symptoms, which should be identified and addressed early to avoid extreme events such as self-harm and suicide [8]. Therefore, attention should be paid to the psychological state of these patients during continuous isolation when SARS-CoV-2 RNA reoccurs.

Regarding the laboratory examination of these six cases, the changes in some indexes at first admission were consistent with previous research reports [9], including normal white blood cells, a decrease in the lymphocyte percentage and absolute value of lymphocytes, an increase in C-reactive protein and erythrocyte sedimentation rate, and normal procalcitonin. Furthermore, at the reoccurrence of SARS-CoV-2 RNA after discharge, the above indexes were all in the normal reference range. The other indexes reflecting liver and kidney function, heart damage, blood coagulation function and other respiratory viruses did not change significantly after the reoccurrence of SARS-CoV-2 RNA, suggesting that the condition of the patients had not deteriorated.

A previous study [4] mentioned that four medical personnel did not cause close-contact infection after the reoccurrence of SARS-CoV-2 RNA. In our study, there were no SARS-CoV-2 RNA-positive cases among the 12 family members who were in close contact with our patients, regardless of whether the family members were previously uninfected or clinically recovered after a previous diagnosis. Moreover, all the patients in our study were farmers or migrant workers, and their awareness of protection during home isolation is low. One study has shown that the lower the RT-PCR Ct value for SARS-CoV-2 RNA detection and amplification indicates the higher the viral load in the samples [10]. In contrast, a high RT-PCR Ct indicates a low viral load, and the significance of the patient as a source of infection also decreases [11]. In this group of cases, the RT-PCR Ct in samples from different body parts after the reoccurrence of SARS-CoV-2 RNA was higher, close to 40. Therefore, although the patients were positive for SARS-CoV-2 RNA, the viral load was low, indicating that they were unlikely to be sources of infection and also it has been reported [12] that no live viruses were isolated after day 8 of onset in a small-size sample.

The time to SARS-CoV-2 RNA-positive conversion in this group ranged from 3 to 15 days after discharge (average 6.5 days). In addition, after all the cases were readmitted to hospital, detection of SARS-CoV-2 RNA in nasopharyngeal, oropharyngeal and anal swabs was performed repeatedly, at least four-times and up to 13-times at intervals of at least 24 h. SARS-CoV-2 RNA could still be detected in different body sites, suggesting that the virus can exist in the multiple sites of human body; furthermore, clearing of the virus varies in different body parts. In addition, the time to SARS-CoV-2 RNA-negative conversion after readmission was 1–16 days (average 7.5 days). Up to now, nasopharyngeal, oropharyngeal and anal swabs have been followed up for 15 days, taken every 5 days, for virus nucleic acid detection and all samples were negative. The human body will produce IgM antibodies for approximately 7 days after infection with SARS-CoV-2 and IgG antibody for approximately 14 days after infection. A study [13] has shown that among 17 confirmed COVID-19 patients, 15 were positive for IgG antibodies when SARS-CoV-2 RNA tests were negative, suggesting the process of disease recovery. Our results showed that viral IgG and IgM antibody tests were positive and negative, respectively, in all cases after the reoccurrence of SARS-CoV-2 RNA, suggesting that these patients were recovering from the disease.

Some researchers [14] proposed that for clinically recovered patients after the epidemic, pulmonary fibrosis may be a problem. In our study, four patients had patchy blurred shadow and ground glass shadow at the first diagnosis, and two patients had no abnormality on chest CT that is consistent with the chest CT findings of previously reported cases [15]. The re-examination of chest CT findings after the reoccurrence of SARS-CoV-2 RNA showed that there were no new lesions in two patients who had no abnormal chest CT findings at the first diagnosis; the other four patients had improved absorption by pulmonary lesions and no pulmonary fibrosis. Therefore, although SARS-CoV-2 RNA was redetected, the imaging findings of the patients were not aggravated; thus, the condition of these patients is unlikely to be serious.

Our study was a descriptive report due to small sample size, but offered detailed follow-up information of different clinical type COVID-19 cases. In addition, serum viral antibodies were not consistently detected when taking samples for the SARS-CoV-2 RNA test from different body sites.

## Conclusion

No worsening outcomes and active transmission to close contacts were found from the readmitted COVID-19 patients with RNA detection back to positivity, irrespective of the age, the coexistense of underlying diseases, the initial clinical classification or the CD4^+^ counts.

**Summary points**

- Some recovered the 2019 coronavirus disease (COVID-19) patients test positive for SARS-CoV-2 RNA within a short time.
- Data on their characteristics and outcomes are limited. Therefore, we described the clinical characteristics and outcomes of recovered COVID-19 patients with the reoccurrence of SARS-CoV-2 RNA.
- Six patients were redetectable and positive for SARS-CoV-2 RNA after discharge from 3 to 15 days.
- The main symptoms, although no fever, included fatigue, dry cough and pharyngeal or chest discomfort, which were generally milder in the repositive period compared with the period of initial infection. Their laboratory indexes were significantly improved compared with the initial infection, and the pulmonary lesions were continuously improving. All close contacts were SARS-CoV-2 RNA-negative.
- The reverse transcriptase-polymerase chain reaction cycle threshold values were all approximated at 40 in the six patients with reoccurrence of SARS-CoV-2 RNA, indicating a lower viral load.
- All close contacts with the six cases with reoccurrence of SARS-CoV-2 RNA were SARS-CoV-2 RNA-negative.
- No worsening outcomes or active transmission to close contacts were found for the readmitted COVID-19 patients with positive SARS-CoV-2 RNA.
